# Simulated microgravity‐induced dysregulation of cerebrospinal fluid immune homeostasis by disrupting the blood–cerebrospinal fluid barrier

**DOI:** 10.1002/brb3.3648

**Published:** 2024-09-11

**Authors:** Jing Yang, Yaoyuan Cui, Juan Zhao, Shiyi Tang, Anqing Wang, Junxiao Wang, Yu Chen, Jilong Luo, Guan Wang, Junhao Yan, Jichen Du, Jiawei Wang

**Affiliations:** ^1^ Beijing Tong Ren Hospital Capital Medical University Beijing China; ^2^ Aerospace Medical Center Aerospace Center Hospital Beijing China; ^3^ Department of Neurology Aerospace Center Hospital Beijing China; ^4^ Institute of Medical Technology Peking University Health Science Center Beijing China; ^5^ Department of Anatomy, Histology and Embryology, School of Basic Medical Sciences Peking University Health Science Center Beijing China

**Keywords:** blood–cerebrospinal fluid barrier, cerebrospinal fluid immune homeostasis, intercellular junction, simulated microgravity

## Abstract

**Background:**

The blood–cerebrospinal fluid barrier (BCSFB) comprises the choroid plexus epithelia. It is important for brain development, maintenance, function, and especially for maintaining immune homeostasis in the cerebrospinal fluid (CSF). Although previous studies have shown that the peripheral immune function of the body is impaired upon exposure to microgravity, no studies have reported changes in immune cells and cytokines in the CSF that reflect neuroimmune status. The purpose of this study is to investigate the alterations in cerebrospinal fluid (CSF) immune homeostasis induced by microgravity and its mechanisms. This research is expected to provide basic data for brain protection of astronauts during spaceflight.

**Methods:**

The proportions of immune cells in the CSF and peripheral blood (PB) of SMG rats were analyzed using flow cytometry. Immune function was evaluated by measuring cytokine concentrations using the Luminex method. The histomorphology and ultrastructure of the choroid plexus epithelia were determined. The concentrations of intercellular junction proteins in choroid plexus epithelial cells, including vascular endothelial‐cadherin (VE‐cadherin), zonula occludens 1 (ZO‐1), Claudin‐1 and occludin, were detected using western blotting and immunofluorescence staining to characterize BCSFB injury.

**Results:**

We found that SMG caused significant changes in the proportion of CD4 and CD8 T cells in the CSF and a significant increase in the levels of cytokines (GRO/KC, IL‐18, MCP‐1, and RANTES). In the PB, there was a significant decrease in the proportion of T cells and NKT cells and a significant increase in cytokine levels (GRO/KC, IL‐18, MCP‐1, and TNF‐α). Additionally, we observed that the trends in immune markers in the PB and CSF were synchronized within specific SMG durations, suggesting that longer SMG periods (≥21 days) have a more pronounced impact on immune markers. Furthermore, 21d‐SMG resulted in ultrastructural disruption and downregulated expression of intercellular junction proteins in rat choroid plexus epithelial cells.

**Conclusions:**

We found that SMG disrupts the BCSFB and affects the CSF immune homeostasis. This study provides new insights into the health protection of astronauts during spaceflight.

## Graphical abstract

Disruption of blood–cerebrospinal fluid barrier mediates altered cerebrospinal fluid immune homeostasis under simulated microgravity: a proposed model. Lymphocytes migrate from the choroid plexus microvasculature to the plexus stroma and then cross the choroid plexus epithelia, where they are locked by tight junctions and enter the cerebrospinal fluid. Simulated microgravity causes disruption of intercellular junctions, allowing more lymphocytes and cytokines to cross the choroid plexus epithelia and enter the cerebrospinal fluid.

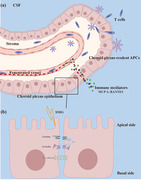

## 1 INTRODUCTION

Human spaceflight missions have experienced a significant surge in recent years. Microgravity poses one of the main challenges for astronauts in space exploration. Extensive research has indicated that microgravity can cause various health problems among astronauts, including bone loss, muscle atrophy, and neurological changes (Ganapathy et al., 2019; Greene et al., 2021). Furthermore, studies have shown that microgravity or simulated microgravity (SMG) can induce neurological conditions such as blood–brain barrier dysfunction (Yan et al., 2021) and choroid plexus epithelial ultrastructural alterations (Gabrion et al., 1995; Masseguin et al., 2001). Several studies have also suggested the possibility of microgravity‐induced neuroimmune responses (Stowe et al., 2003). It should be noted that neuroimmune dysfunction is associated with neurological disorders, including increased lymphocytes in the cerebrospinal fluid (CSF), altered cytokine levels, and downregulated intercellular junction proteins like TJs and AJs in the choroid plexus epithelia (Coisne & Engelhardt, 2011; Shen et al., 2022). The blood–cerebrospinal fluid barrier (BCSFB) is formed by choroid plexus epithelial cells (Solár et al., 2020). Choroid plexuses are distributed in the lateral, third, and fourth brain ventricles, floating in the CSF space (Zuba et al., 2022). In particular, the central core of the choroid plexus stroma is filled with immune cells (Gelb & Lehtinen, 2023). Although immune cells and cytokines can move from the capillaries to the choroid plexus stroma, they cannot reach the CSF through the choroid plexus epithelia due to the presence of tight junctions (TJ), including zonula occludens 1 (ZO‐1) (Uchida et al., 2019), occluding, Claudin‐1 (Steinemann et al., 2016), and adherens junctions (AJ) like vascular endothelial‐cadherin (VE‐cadherin) (Shen et al., 2022). The integrity of the choroid plexus intercellular junctions plays an integral part in the maintenance of CSF immune homeostasis (Shen et al., 2022). CSF, produced primarily by choroid plexus epithelia, serves as a reflection of numerous biochemical and cellular events in the brain parenchyma (Mapunda et al., 2022). Several studies have suggested that T cells enter the CSF through the choroid plexus epithelia (Schlager et al., 2016). In neurological disorders such as Lyme neuropathy, bacterial meningitis, viral meningitis, clinical isolation syndrome, and relapsing multiple sclerosis, immune dysregulation of the CSF is characterized by significant changes in the proportion of CD4 T (CD3^+^/CD4^+^) and CD8 T (CD3^+^/CD8^+^) immune cells as well as cytokine levels in the CSF (Schröder et al., 2018). Although studies on the impact of in‐orbit conditions on the production of CSF have been conducted previously (Gabrion et al., 1995), research on the immune status of CSF under microgravity is limited. Studies on how periods of microgravity affect the immune equilibrium of CSF are also lacking. Previous research has only reported that in the very early stages (< 180 min), the microvilli of the choroid plexus in rats become longer and thinner, without changes observed in Claudin‐1 and ZO‐1 (Masseguin et al., 2001). The effects of microgravity over different periods on the expression of intercellular connections in the choroid plexus have not been addressed. The role of the BCSFB in altering the CSF immune balance has also not been explored.

This paper aims to investigate the effects of microgravity on CSF immune homeostasis and the relationship between varying durations of microgravity exposure and CSF stability. It also explores the initial hypothesis that BCSFB integrity disruption could be a potential mechanism behind CSF immune homeostasis dysregulation. The study first examined how various immune markers in CSF and peripheral blood (PB) changed during periods of simulated microgravity (SMG). It then analyzed the correlation between the trends of changes in neuroimmune and peripheral immune indicators. Finally, the integrity of the BCSFB was assessed by evaluating the expression of junction proteins in the choroid plexus epithelial cells. It is expected to supply basic data for brain protection of astronauts during spaceflight.

## 2 MATERIALS AND METHODS

### 2.1 Animals and experimental design

Sixty SPF SD rats (male) weighing 280–320 g were obtained from the National Institute for Food and Drug Control. All rats (2–4 months old) were housed under hygienic laboratory conditions with temperature (21 ± 2°C) and humidity (60 ± 10%) on a 12‐h day/light cycle, with food and water available ad libitum. The Peking University Aerospace Clinical College Animal Care and Use Committee approved this study (CR‐20200826‐NSFC‐01). The rats were randomly assigned into the 0° control group (7d‐NG, 14d‐NG, 21d‐NG, 28d‐NG, 35d‐NG) and the −30° hindlimb unloading group (7d‐SMG, 14d‐SMG, 21d‐SMG, 28d‐SMG, 35d‐SMG), resulting in a total of 10 groups with six rats in each group (*n* = 6). In the −30° hindlimb unloading groups, the rats’ forelimbs could touch the ground while the hind limbs were elevated, resulting in a head‐down tilt of 30°. In the 0° control groups, both the front and hind limbs of the rats could touch the ground, and only the tails were suspended in the organic transparent glass modeling cage.

### 2.2 Collection of rats’ CSF and PB

Rats were anesthetized with 5% isoflurane, maintained in oxygen with 2% isoflurane, and fixed in a prone position on the stereotaxic apparatus (ZSDC Technology Ltd., BJ, China) with the head fixed and neck skin prepared. Approximately 100 µL of CSF per rat was aspirated through the large occipital foramen using a 1 mL syringe (BD, NY, USA). Rats were anesthetized by removing gas and were injected intraperitoneally with 3% pentobarbital sodium (55 mg/kg). After deep anesthesia, the abdominal cavity was opened, and blood was taken through the heart and placed in an anticoagulation tube. The blood collected was then centrifuged (1500 rpm, 8 min) and stored at −80°C for subsequent Luminex analysis. Cells were precipitated, treated with erythrocyte lysate (BD, NY, USA), and centrifuged for flow cytometry analysis.

### 2.3 Flow cytometry analyses of rats’ CSF and PB

The samples were divided into two tubes. Tube 1 was supplemented with APC/cyanine7 CD45, PE CD172a (SIRPα), APC CD43, FITC CD3, and PE/Cy7 CD45RA antibody. Tube 2 was supplemented with FITC CD3, APC/cystine7 CD45, APC CD4, PerCP CD8a, and PE anti‐rat CD161 antibody. Vortex and mix well, then incubate for 15 min protected from light. The cells were then incubated in hemolysin for 10 min, centrifuged to remove the supernatant, and again added to the staining buffer. Cells were analyzed on BD FACS Cantoll using FACS DiVa 8.0 software (BD, NY, USA).

### 2.4 Luminex analyses of rats’ CSF and PB

PB/CSF was collected at 7d‐/14d‐/21d‐/28d‐/35d‐SMG. All samples from rats were stored at −80 °C without repetitive freezing and thawing. Cytokine levels were determined using the Luminex liquid suspension chip detection method by Wayen Biotechnologies (Shanghai, China). Briefly, samples were incubated in 96‐well plates with microbeads for 30 min and then incubated with the corresponding detection antibody for 30 min. Subsequently, streptavidin‐PE was added to each well for 10 min of incubation. The values were read by the calibrated Bio‐Plex System (Bio‐Rad).

### 2.5 Histomorphological observation

The rat brain tissue was partitioned using rat brain molds, and the tissue near the hippocampus was fixed in 4% paraformaldehyde, and pathological sections and transmission electron microscopy ultrathin sections were produced after fixation. Pathological sections were prepared at the Department of Pathology, Aerospace Central Hospital, with a section thickness of 4 µm, HE staining, and final histological images were acquired using a KF‐PRO‐005‐EX digital pathology scanner (KFBIO Ltd., ZJ, China). Transmission electron microscopy sections were prepared at the School of Science, Beijing Jiaotong University, with a section thickness of 50 nm, and the ultrastructure of the choroid plexus was observed using a transmission electron microscopy instrument (JEOL Ltd., Tokyo, Japan) after fabrication.

### 2.6 Western blotting

Choroid plexuses of rats were homogenized using a tissue lyser (QIAGEN, Hilden, Germany) to extract total protein. The electrophoresis conditions were a constant voltage of 80 V for 90 min. The transfer conditions were constant flow 200 mA, 120 min. To block nonspecific bindings, the membranes were incubated for 2 h. Subsequently, the membranes were incubated with the primary antibody for 14 h and the secondary antibody for 2.5 h at 25°C. Finally, membranes were developed to analyze the gray values of target proteins.

### 2.7 Immunofluorescence staining

The expression levels of VE‐cadherin, ZO‐1, Claudin‐1, and occludin were assessed using immunofluorescence analysis. Primary antibodies: rabbit anti‐VE‐cadherin (YT5611, dilution 1:200), ZO‐1 (ab190085, dilution 1:200), Claudin‐1 (ab15098, dilution 1:200), and occludin (ab216327, dilution 1:200). Secondary antibodies: donkey anti‐rabbit Alexa Fluor 488 (ab150073, dilution 1:500) and donkey anti‐goat Alexa Fluor 555 (ab150130, dilution 1:1000). To visualize the cell nuclei, a DAPI‐containing blocker (Solarbio, Beijing, China) was applied to the sections. Finally, sections were observed using an Olympus IX73‐F22FL/PH fluorescence contrast microscope equipped with cellSens image processing software (Olympus, Tokyo, Japan).

### 2.8 Statistical analysis

All data were analyzed by GraphPad Prism 10.1 software, presented as mean ± standard error of the mean (SEM). Data were analyzed with an analysis of variance (ANOVA) test followed by the Sidak's test. ANOVA was used to evaluate the interaction effects between the factors. Results were considered statistically significant at *p‐*value of < .05. Following significant ANOVA results, Sidak's multiple comparisons test was applied to determine specific group differences. The α error was set at.05.

## 3 RESULTS

### 3.1 Alterations of T cells in CSF under SMG

**FIGURE 1:**
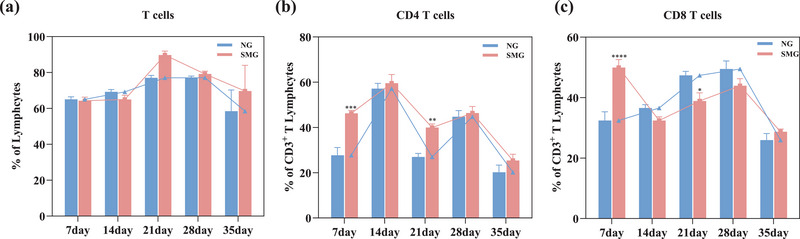
Simulated microgravity affected the proportions of T cells in rat cerebrospinal fluid. (a) Flow cytometry analysis of T cells, (b) CD3^+^/CD4^+^ T cells, and (c) CD3^+^/CD8^+^ T cells. Statistical analysis involved the use of a two‐way ANOVA, followed by the Sidak's test. * indicates a significant difference compared with the NG group, *p < .05, **p < .01, ***p < .001, ****p < .0001. Data were described as mean ± SEM (*n* = 6 per group).

We first analyzed the change of immune cells in the CSF under SMG. CSF samples were exacted from the cistern magnum under SMG of 7d, 14d, 21d, 28d, and 35d. Afterward, flow cytometry analysis was completed within 2 h after sampling. Compared with the NG group, CD4 and CD8 showed significant differences under 7/21 days of the SMG group. CD4 T cells exhibited a notable increase of 67.08% (Figure 1b, *p *< .001) and CD8 T cells had increased significantly by 54.02% under 7d‐SMG (Figure 1c, *p *< .0001). CD4 T cells were increased significantly by 48.05% under 21d‐SMG (Figure 1b, *p *< .01), while CD8 showed an evident decline of 17.91% (Figure 1c, *p *< .05).

In addition to comparing the SMG group with the NG group, we have also analyzed immune cell responses over varying durations of SMG. As time progresses, the pattern of changes in the proportion of T cells in the CSF of rats in both the SMG and NG groups is similar, exhibiting an initial increase followed by a decrease. The effect of 21d‐SMG had the greatest impact on the proportion of T cells, manifesting as an increase of 16.52% (Figure 1a). The trend of changes in the proportion of CD4 T cells in both the SMG and NG groups is relatively parallel, both showing an initial increase followed by a decrease (Figure 1b). Notably, 7 days and 21 days of SMG exposure are points of interest for CD4 T cells, as SMG leads to a significant increase in the proportion of CD4 T cells. During the 14‐ to 35‐day SMG period, the pattern of CD8 T cell fluctuations in the CSF of rats was comparable between the SMG and NG groups. Notably, there was a significant decrease in the proportion of CD8 T cells by 17.92% at 21d‐SMG. Therefore, it appears that longer durations of SMG, such as 21 days, are more likely to substantially affect the ratio of immune cells in the CSF than other SMG exposure periods.

### 3.2 Alterations of immune cells in PB under SMG

**FIGURE 2:**
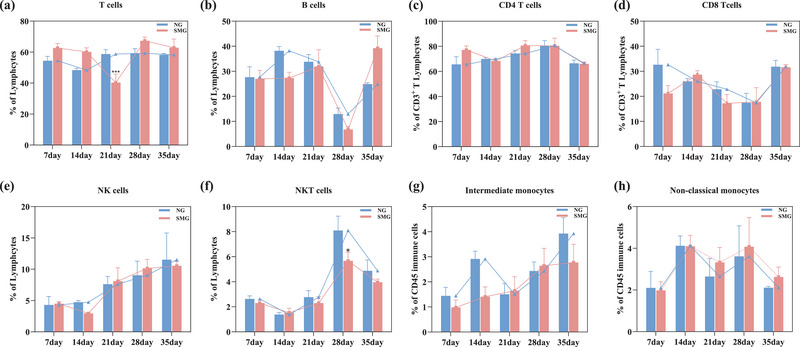
Simulated microgravity affected the proportion of immune cells in rat peripheral blood. (a) Flow cytometry analysis of CD45^+^/CD3^+^ T cells, (b) CD3^−^/CD45RA^+^ B cells, (c) CD3^+^/CD4^+^ T cells, (d) CD3^+^/CD8^+^ T cells, (e) CD3^−^/CD161a^+^ NK cells, (f) CD3^+^/CD161a^+^ NKT cells, (g) CD172a^+^/CD43 low intermediate monocytes, and (h) CD172a^+^/CD43 high nonclassical monocytes. Statistical analysis involved the use of a two‐way ANOVA, followed by the Sidak's test. * indicates a significant difference compared with the NG group, *p < .05, ***p < .001. Data were described as mean ± SEM (*n* = 6 per group).

To investigate the impact of SMG on systemic immunity, The proportions of immune cells in rat PB were examined (7d, 14d, 21d, 28d, and 35d). The immune cell subsets analyzed included T cells (CD45^+^/CD3^+^), CD4 T cells (CD3^+^/CD4^+^), CD8 T cells (CD3^+^/CD8^+^), B cells (CD3^−^/CD45RA^+^), NK cells (CD3^−^/CD161a^+^), NKT cells (CD3^+^/CD161a^+^), intermediate monocytes (CD172a^+^/CD43 low) and nonclassical monocytes (CD172a^+^/CD43 hi) in rat PB. T cells exhibited significant decreases of 26.63% under 21d‐SMG (Figure 2a, *p *< .001). NKT cells were decreased significantly by 38.96% under 28d‐SMG (Figure 2f, *p *< .05).

With prolonged exposure to SMG, the trajectory of T and B cell variations in peripheral blood (PB) maintained a relatively steady course, registering a noteworthy decline only at the 21d‐ and 28d‐SMG (Figure 2a and b). The ratios of CD4 to CD8 T cells remained largely unchanged as the duration of SMG was extended. Throughout the 14d–35d‐SMG period, the dynamic trends in the NG and SMG groups progressed in tandem (Figure 2c and d). During the 14d–28d‐SMG, even though there was a general upward trend in NK and NKT cells, a significant uptick in the proportion of NKT cells in PB was exclusively observed on the 28th day under SMG (Figure 2e and f). The variation in both classes of monocytes under NG and SMG conditions displayed analogous patterns and was not markedly influenced by prolonged SMG exposure (Figure 2g and h). In sum, the longer SMG cycles (exceeding 21 days) more clearly demonstrated their effects on the distribution of peripheral immune cells as the SMG duration increased.

### 3.3 Alterations in cytokines in CSF under SMG

**FIGURE 3:**
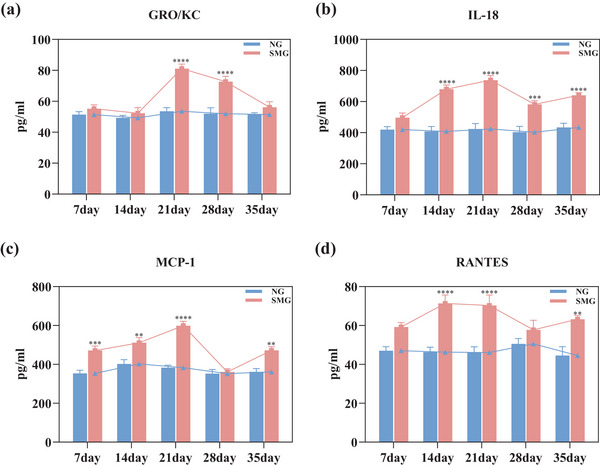
Protein levels of cytokines in the cerebrospinal fluid. Cerebrospinal fluid was collected from 7d‐, 14d‐, 21d‐, 28d‐, and 35d‐SMG rats. (a–d) The levels of GRO/KC, IL‐18, MCP‐1, and RANTES were measured by Luminex. Changes in GRO/KC, IL‐18, MCP‐1, and RANTES cytokines. Statistical analysis involved the use of a two‐way ANOVA, followed by the Sidak's test. * indicates a significant difference compared with the NG group, **p < .01, ***p < .001, ****p < .0001. Data were described as mean ± SEM (*n* = 6 per group).

To assess specific changes in neuroimmune function under SMG, the concentrations of 23 cytokines in the CSF were analyzed. Among them, four pro‐inflammatory factors, namely GRO/KC, IL‐18, MCP‐1, and RANTES, exhibited significant elevations in the CSF. GRO/KC protein levels had increased by 51.49 with 21d‐SMG and 39.82% with 28d‐SMG (Figure 3a, both *p *< .0001). IL‐18 had increased by 66.47% under 14d‐SMG, 74.08% under 21d‐SMG, 44.24% under 28d‐SMG, and 47.5% under 35d‐SMG (Figure 3b, *p *< .0001, *p *< .0001, *p *< .001, and *p *< .0001, respectively). MCP‐1 showed a significant increase of 33.28% under 7d‐SMG, 27.02% under 14d‐SMG, 56.47% under 21d‐SMG, 30.4% under 35d‐SMG (Figure 3c, *p *< .001, *p *< .01, *p *< .0001, and *p *< .01, respectively). RANTES demonstrated significant increases of 54.02% under 14d‐SMG, 52.59% under 21d‐SMG, and 41.94% under 35d‐SMG (Figure 3d, *p *< .0001, *p *< .0001, and *p *< .01, respectively).

With the extension of SMG exposure, the overall trend observed in the levels of four cytokines (GRO/KC, IL‐18, MCP‐1, RANTES) within the CSF was indicative of an increase. Notably, during the 7–21 days of SMG, there was a sharp increase in cytokine levels, whereas from 21 to 35 days of SMG, the increase in cytokine levels was more gradual. Therefore, it is evident that the concentration of certain inflammatory factors in the CSF reaches its peak at 21d‐SMG.

### 3.4 Alterations in cytokines in PB under SMG

**FIGURE 4:**
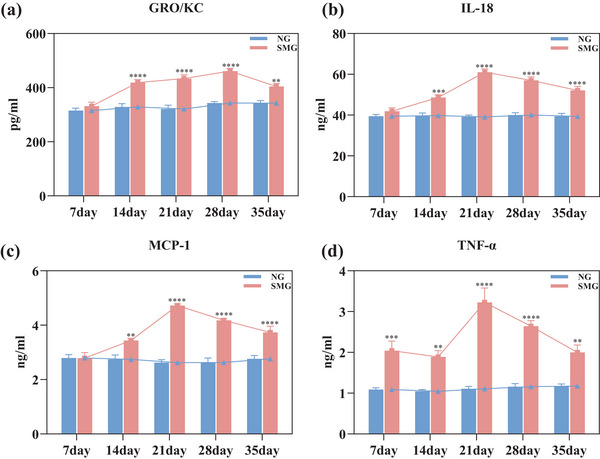
Protein levels of cytokines in serum peripheral blood. Blood was collected from the heart of 7d‐, 14d‐, 21d‐, 28d‐, and 35d‐SMG rats. (a–d) The levels of GRO/KC, IL‐18, MCP‐1, and TNF‐α were measured by Luminex. Changes in GRO/KC, IL‐18, MCP‐1, and TNF‐α cytokines. Statistical analysis involved the use of a two‐way ANOVA, followed by the Sidak's test. * indicates a significant difference compared with the NG group, **p < .01, ***p < .001, ****p < .0001. Data were described as mean ± SEM (*n* = 6 per group).

To further evaluate peripheral immune function, the levels of 23 cytokines were detected. Likewise, GRO/KC, IL‐18, MCP‐1, and TNF‐α, exhibited significant elevations in PB under SMG. GRO/KC protein levels in PB had increased by 27.46% with 14d‐SMG, 35% with 21d‐SMG, 34.27% with 28d‐SMG, and 18.03% with 35d‐SMG (Figure 4a, both *p *< .0001). IL‐18 had increased significantly by 22.38% under 14d‐SMG, 56.01% under 21d‐SMG, 42.42% under 28d‐SMG, and 32.27% under 35d‐SMG (Figure 4b, *p *< .001, *p *< .0001, *p *< .0001, and *p *< .0001, respectively). MCP‐1 was increased significantly by 25.38% under 14d‐SMG, 80.02% under 21d‐SMG, 58.82% under 28d‐SMG, and 35.21% under 35d‐SMG (Figure 4c, *p *< .01, *p *< .0001, *p *< .0001, and *p *< .0001, respectively). TNF‐α exhibited significant increases of 87.6% under 7d‐SMG, 80.57% under 14d‐SMG, 191.24% under 21d‐SMG, 127.96% under 28d‐SMG, and 70.21% under 35d‐SMG (Figure 4d, *p *< .001, *p *< .01, *p *< .0001, *p *< .0001, and *p *< .01, respectively).

As the duration of SMG exposure increases, the levels of four factors in the PB—GRO/KC, IL‐18, MCP‐1, and TNF‐α—generally show an upward trend. IL‐18, MCP‐1, and TNF‐α concentrations peak at 21d‐SMG, while the concentration of GRO/KC reaches its maximum at 28d‐SMG (Figure 4a). This indicates that the 21‐ to 28‐day SMG period has a more substantial impact on peripheral immunity compared to other SMG durations.

### 3.5 Analysis of immune marker variation trends in PB and CSF

**FIGURE 5:**
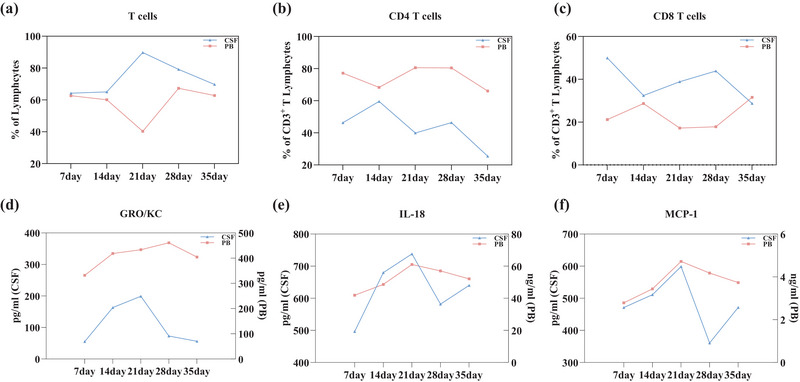
Immune marker variation trends in peripheral blood and cerebrospinal fluid. (a–f) The vertical axis *Y* of the line chart represents the difference between the SMG group and the NG group.

Our analysis reveals that during longer SMG cycles (≥21 days), the trends of immune cells (T cells, CD4 T cells, CD8 T cells) in both CSF and PB are parallel (Figure 5a–c). The cytokine analysis results indicate that as the SMG cycle lengthens, the changes in three cytokines (GRO/KC, IL‐18, MCP‐1) in both cerebrospinal fluid and peripheral blood continue to increase (Figure 5d–f).

The trend of changes in T Cells, CD4 T Cells, CD8 T Cells, GRO/KC, IL‐18, and MCP‐1 in cerebrospinal fluid and peripheral blood was observed.

### 3.6 Histomorphological of brain CP under SMG

**FIGURE 6:**
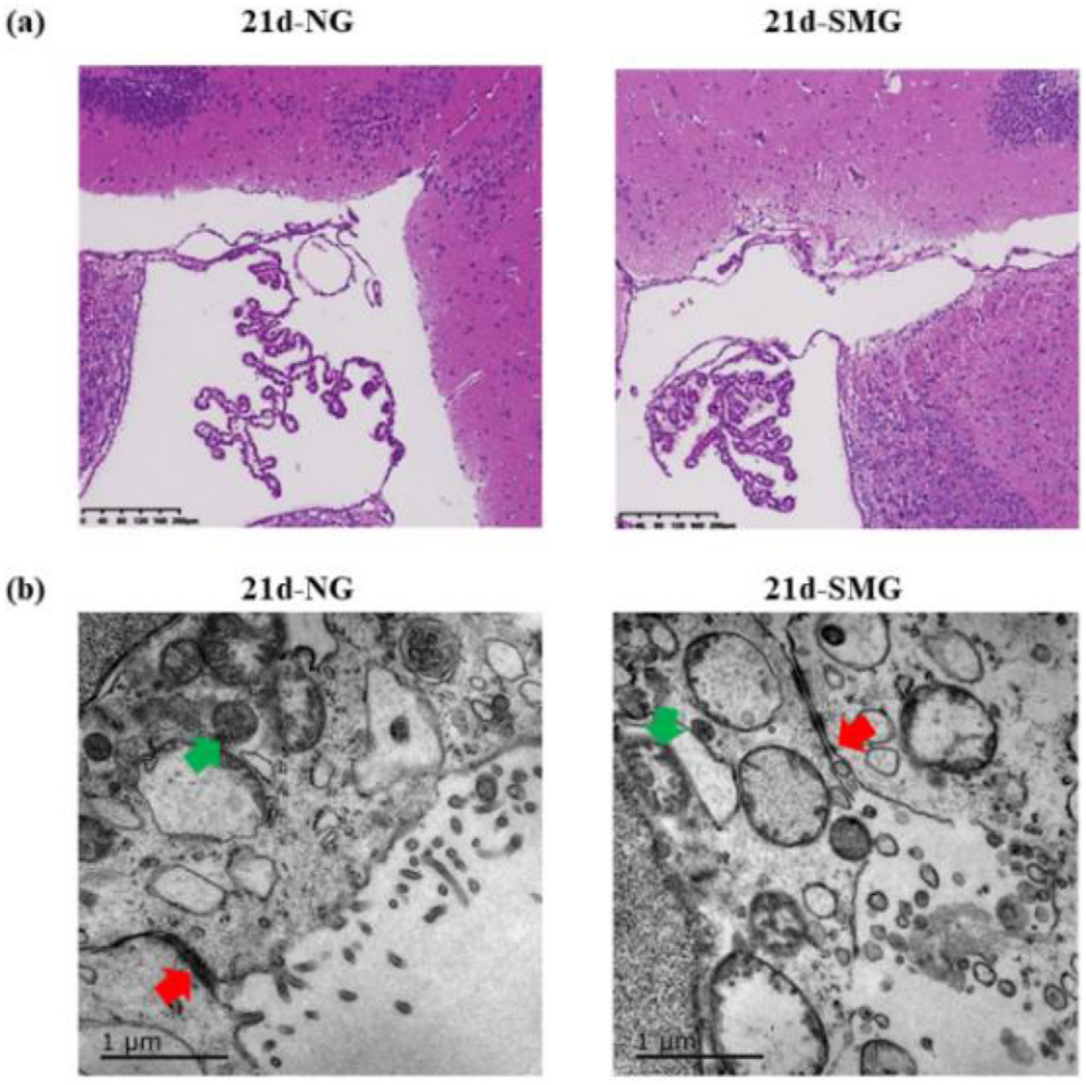
Simulated microgravity (SMG) damaged the ultrastructure of the choroid plexus. (a) HE staining in the choroid plexus of the NG and SMG groups (Scale bar is 100 µm, *n* = 6). (b) Ultrastructure of choroid plexus as observed by transmission electron microscopy in the NG and SMG groups. Red arrows point to the breakdown of intercellular junctional structures under SMG, and green arrows point to unclear mitochondrial membrane structures under SMG (Scale bar is 1 µm, *n* = 6).

Based on the previous results, we selected 21d‐SMG for morphological observation of the CP tissue. The organization of the CP under 21d‐SMG was observed. No obvious differences were observed in the SMG group with HE (Figure 6a). However, ultrastructural observations revealed loosened intercellular junctions under SMG, unclear mitochondrial bilayers, and no intact cristae of endosome formation (Figure 6b).

### 3.7 Effect of SMG on the expression of intercellular junction proteins in rat brain CP

**FIGURE 7:**
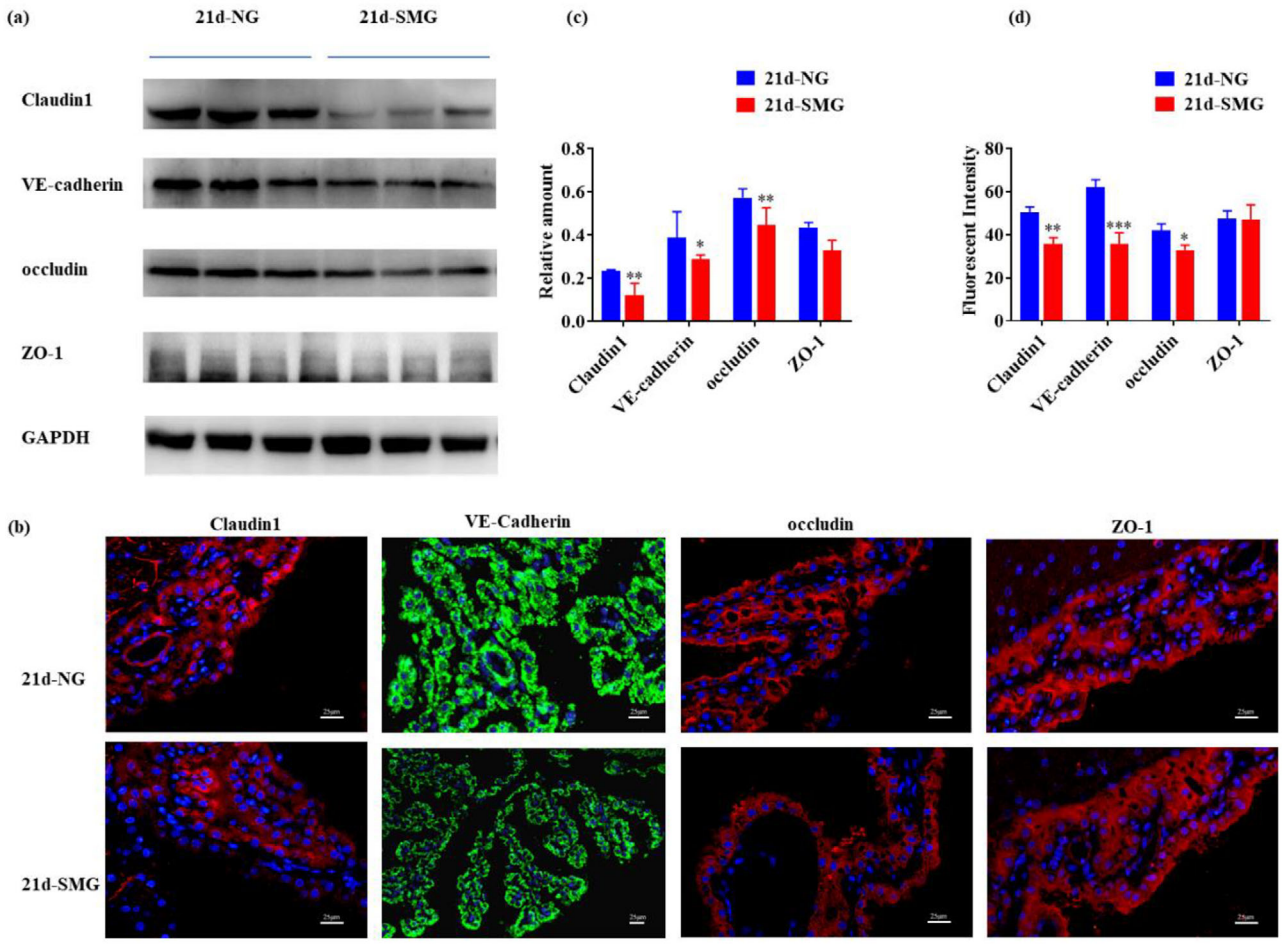
Simulated microgravity (SMG) induced intercellular junction protein levels in the choroid plexus. (a) The protein levels of Claudin‐1, VE‐cadherin, occluding, and ZO‐1 determined by Western blotting (*n* = 6). (b) Immunofluorescence detection of Claudin‐1, VE‐cadherin, occludin, and ZO‐1 in choroid plexus tissues of SMG group rats (scale bar: 25 µm). (c) The relative amount of protein in (a). (d) The fluorescence intensity of the protein in (b). * indicates significant difference compared with the NG group, *p < .05, **p < .01, ***p < .001, one‐way ANOVA was used. Data were described as mean ± SEM (*n* = 6 per group).

To explore the impact of SMG on intercellular junctions, we assessed the protein expression of three TJ proteins (ZO‐1, ocludin, Claudin‐1) and one AJ protein (VE‐adherin) in choroid plexus using Western blotting (Figure 7a). Intercellular junction proteins play a crucial role in maintaining the integrity of the BCSFB (Brkic et al., 2015). The Western blotting results demonstrated a significant reduction in the expression of Claudin‐1, VE‐cadherin, and occludin in the choroid plexus of SMG rats compared to the NG group (Figure 7a and c, *p *< .01, *p *< .05, *p *< .01, respectively). Furthermore, immunofluorescence analysis confirmed a significant decrease in the expression of Claudin‐1, VE‐cadherin, and occludin in the choroid plexus of SMG rats (Figure 7b and d, *p *< .01, *p *< .001, *p *< .05, respectively).

## 4 DISCUSSION

Previous research has demonstrated a decrease in CSF secretion under microgravity conditions (Gabrion et al., 1995). Our study has made the novel discovery that the proportion of CD4 T cells in CSF exhibits a time‐dependent increase from 7 to 14 days of SMG condition. Similarly, the proportion of CD8 T cells shows a time‐dependent increase from 14 to 28 days of SMG. CD4 T cells differentiate into different subsets of helper effectors, secrete relevant cytokines, and play a role in immune responses (Zhu et al., 2010). On the other hand, CD8 T cells have the ability to specifically identify and eliminate target cells (Barry & Bleackley, 2002).

Additionally, this study has observed a significant decrease in the proportion of T cells in PB after 21 days of SMG and a significant decrease in NKT cell proportion after 28 days, with no significant changes at other time points. Research has shown that after 10–15 days of spaceflight, there would be no significant changes in the cell numbers of white blood cell subsets (granulocytes, monocytes, lymphocytes) in peripheral blood, nor in lymphocyte subsets (T cells, B cells, NK cells), including T cell subsets (CD4, CD8) (Crucian et al., 2013). NK cell function has been found to be more vulnerable to impairment in astronauts undergoing a 3‐month spaceflight mission for the first time (Bigley et al., 2019). Another study has evaluated the phenotypic and functional changes in the immune system of astronauts participating in a 6‐month spaceflight, showing a decrease in T cell numbers (Crucian et al., 2015).

In order to fully assess immune function, protein levels of 23 cytokines/chemokines have been quantified in both PB and CSF. Previous studies have demonstrated alterations in the cytokine/chemokine profile in PB, indicating changes in peripheral immune function in SMG (Li et al., 2021). In line with these findings, our study detected significant increases in GRO/KC, IL‐18, MCP‐1, and TNF‐α levels in rats' PB under SMG (Figure 4a–d), these cytokine/chemokine mediate inflammation, immune diseases, and metabolic diseases (Dinarello et al., 2013). Furthermore, we have observed significant changes in GRO/KC, IL‐18, and MCP‐1 levels in both CSF and PB (Figures 3 and 4). The alterations in GRO/KC levels have occurred later in the CSF compared to PB. GRO/KC, an inflammatory chemokine, is involved in processes such as the induction of angiogenesis and neutrophil recruitment (Korbecki et al., 2022). Elevated levels of GRO/KC in CSF have been reported in patients with certain neuroimmune diseases (Zwijnenburg et al., 2003). In addition, we have identified a remarkable lift of RANTES and MCP‐1 cytokines in the CSF. RANTES serves as a chemokine for T cells and monocytes, primarily acting in the recruitment of leukocytes to spots of inflammation (Appay & Rowland‐Jones, 2001). The substantial elevation of GRO/KC, IL‐18, RANTES, and MCP‐1 in the CSF strongly suggests that SMG induces neuroimmune changes (Figure 4).

**FIGURE 8:**
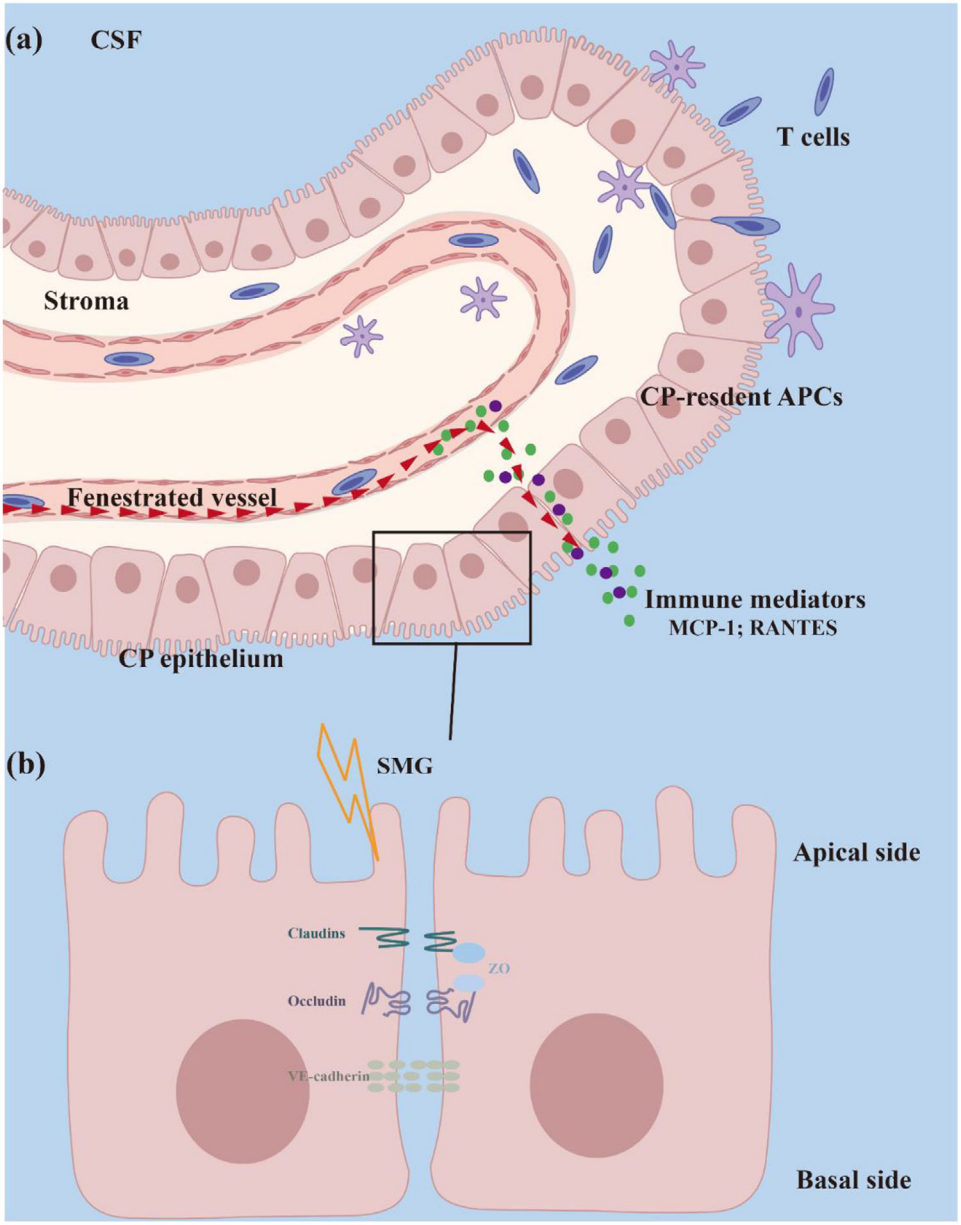
Disruption of BCSFB mediates altered cerebrospinal fluid immune homeostasis under simulated microgravity: a proposed model. (a) Lymphocytes migrate from the choroid plexus microvasculature to the plexus stroma and then cross the choroid plexus epithelia, where they are locked by a tight junction and enter the choroid plexus. Simulated microgravity causes disruption of intercellular junctions, allowing more lymphocytes and cytokines to cross the choroid plexus epithelia and enter the cerebrospinal fluid. (b) Anatomy of intercellular junctions of the choroid plexus epithelia. Tight junction proteins, such as occludin, Claudin‐1, and ZO‐1, and adherens junction proteins, such as VE‐cadherin, form intercellular junctions between choroid plexus epithelial cells and limit exchange of substances between blood and CSF.

BCSFB performs essential functions for brain vitality, including the removal of neuronal/glial waste, and the transport of nutrients, hormones, neurotransmitters, and neuropeptides to the CNS (de Lange, 2004). It is notable that lymphocytes can also migrate across the BCSFB in the choroid plexus. Normally, lymphocytes can migrate from the choroid plexus microvasculature to the plexus stroma and then are stopped by the choroid plexus epithelia where TJs block the crossing path entering the CSF. Since our histological and ultrastructural observations of choroid plexus have shown that 21d‐SMG induced ultrastructural changes (Figure 6), we have selected the 21d‐SMG as the time node and then examined the expression of intercellular junctions in the choroid plexus epithelia. As a result, we have discovered that 21d‐SMG induced a declined level of junction protein (Figure 7). In conclusion, we propose the hypothesis that SMG may increase the probability of the cytokines and T cells in the choroid plexus stroma entering the CSF, by damaging the BCSFB (Figure 8). Our study has observed prolonged SMG‐induced damage to the BCSFB, which is supported by previous studies (Gabrion et al., 1995; Gabrion et al., 1996; Masseguin et al., 2001). Also, SMG has been reported to cause damage to the BBB by affecting the cytoskeleton, which is consistent with our findings (Yan et al., 2021).

Although this project does not further explore how BCSFB is damaged, we speculate that it is related to MCP‐1. MCP‐1 is mainly secreted by astrocytes in the brain, and prolonged SMG causes an increase in the number of astrocytes (Mao et al., 2020). MCP‐1 can further regulate intercellular junctions by binding to epithelial cell surface receptors to activate Rho‐A/MLC pathways and affect the cytoskeleton (Stamatovic et al., 2003). Meanwhile, SMG‐mediated cytoskeletal rearrangement via MLC has been also verified (Wang et al., 2021). Thus, SMG may change neuroimmune function by disrupting intercellular junctional structures by upregulating MCP‐1 levels. Therefore, SMG may mediate BCSFB injury by disrupting intercellular junctional structures by upregulation of MCP‐1 levels.

The relationship between microgravity, stress, and immunity has been a widely discussed topic in the scientific community. Among the numerous factors that may affect immunity during spaceflight, stress may play a significant role (Sonnenfeld, 1999). It has been reported that stress plays a significant role in affecting astronauts' immune markers, including increased levels of sympathetic nervous system activity and alterations in stress hormones (Meehan, et al., 1993; Tipton, et al., 1996). We believe that stress is an unavoidable link between microgravity and immune responses, but the factors that induce immune reactions induced by spaceflight remain largely to be elucidated.

Regarding the specificity of the observed responses to microgravity, we delved into how the changes in immune markers in CSF and PB indicate the body's response to the microgravity environment. We cited studies conducted under actual spaceflight conditions that investigated similar immune responses, supporting the view that these responses are not merely stress responses but are specifically related to microgravity. For example, the shift in the proportion of T cells in PB and the increase in levels of cytokines such as GRO, MCP‐1, and TNF‐α have been documented in astronauts (Crucian, et al, 2014; Garrett‐Bakelman, et al, 2019), further validating the specificity of these immune changes to microgravity.

Research on changes in immune function caused by microgravity has mainly focused on changes in peripheral immune function (Crucian et al., 2013) and mucosal immune function (Li et al., 2021), with very few reports on CSF. Our study is the first to focus on the altered neuroimmune in rats under SMG through changes in CSF cell subsets and immune factors. We have found that the response times of different immune cells and factors to SMG are different, and peripheral immunity is preceded by neuroimmunity. In addition, we have explored the potential relevance between SMG‐induced neuroimmune alteration and the condition of intercellular junctions in the choroid plexus epithelia.

Our study still has limitations; at different time points, the responses shown by the control group fluctuated. We believe that factors contributing to this variability may include the characteristics of biological samples, sample sizes, and environmental changes. We acknowledge that we could not control or consider these factors fully in this study. Otherwise, our study's exploration into the mechanisms behind neuroimmune changes was only preliminary, examining the perspective of BCSFB barrier disruption. A deeper understanding of the mechanisms of BCSFB damage could perhaps better explain the neuroimmune changes under microgravity. With these modifications, we hope to clearly communicate this work's limitations and provide useful insights for future research.

## 5 CONCLUSION

We found that SMG disrupts the BCSFB and affects the CSF immune homeostasis. This study provides new insights into the health protection of astronauts during spaceflight.

## AUTHOR CONTRIBUTIONS

**Jing Yang**: Writing—original draft; writing—review and editing. **Yaoyuan Cui**: Writing—original draft; writing—review and editing. **Juan Zhao**: Writing—review and editing. **Shiyi Tang**: Writing—review and editing. **Anqing Wang**: Methodology. **Junxiao Wang**: Data curation. **Yu Chen**: Supervision. **Jilong Luo**: Methodology. **Guan Wang**: Funding acquisition. **Junhao Yan**: Supervision. **Jichen Du**: Resources; writing—review and editing; supervision; funding acquisition. **Jiawei Wang**: Resources; writing—review and editing; supervision.

## CONFLICT OF INTEREST STATEMENT

The authors declare that the research was conducted without any commercial or financial relationships that could potentially create a conflict of interest.

### PEER REVIEW

The peer review history for this article is available at https://publons.com/publon/10.1002/brb3.3648.

## Data Availability

The raw data supporting the conclusion of this article will be made available by the authors, without undue reservation.
